# What are the unmet support needs of women with a known BRCA1/2 mutation?

**DOI:** 10.1186/1897-4287-10-S2-A54

**Published:** 2012-04-12

**Authors:** V White, A Farrelly, B Meiser, M Jefford, M Young, I Winship, S Ieropoli, J Koehler

**Affiliations:** 1 Cancer Council Victoria, Australia

## Background

Women known to carry a BRCA1/2 mutation have to make complex decisions regarding the management of their increased cancer risk, such as whether or not to undergo risk-reducing surgery and how to communicate this risk information to family members. These women may face a unique set of supportive care needs, although unlike the situation for breast and other cancer patients, few supportive care services for mutation carriers exist outside the Familial Cancer Centres. The aim of this presentation is to describe the unmet support needs of women with a known BRCA 1/2 mutation volunteering for a randomised control trial (RCT) testing the effectiveness of a telephone based peer support program.

## Methods

Participants were recruited through Familial Cancer Centres in Victoria, New South Wales and South Australia. Women between the ages of 18-70, who tested positive for a BRCA1/2 mutation within the last 4 years were eligible for participation in the study. Participants were recruited as part of an ongoing RCT. Women completed a baseline survey after enrolling in the study and prior to randomisation. The baseline survey included a 16-item scale assessing unmet support needs, such as dealing with fears about developing cancer, wanting reassurance that feelings are normal, and talking to others in a similar situation. Women were asked to indicate their level of need in the past month on each item using a 5-point likert scale.

## Results

216 participants have completed the baseline survey to date (response rate of 46%). The average age of participants was 47.2 years (SD:13.6) and 44.0% (n=95) had a previous diagnosis of breast or ovarian cancer. The mean time since notification of carrier status was 2.5 years (SD=2.1).

The average number of moderate to very high needs among all participants was 4.7 (S.D 4.6). Twenty six percent (n=52) of participants had no moderate to very high needs, while 23.1% (n=46) had a moderate to very high need on eight or more items. The most commonly endorsed moderate to very high needs were: “dealing with uncertainty about the future” (42%), “dealing with the impact a faulty gene has had on your family” (40%), and “dealing with fears about developing cancer” (39%). The least endorsed items were: “dealing with insurance issues” (61% indicated no need), “dealing with feelings of isolation” (53% no need) and “obtaining more information about your risk for breast cancer” (53%). The average number of moderate to very high unmet needs was not related to previous history of cancer (P=0.94), having children (p=0.87) or time since receiving BRCA1/2 mutation status results (P=0.36).

## Discussion

Many women with a known BRCA 1/2 mutation have unmet supportive care needs that are not currently being addressed. Level of unmet need is not related to time since receiving test results, history of cancer or having children. The RCT trial will assess whether a telephone-based peer support program meets these unmet needs.

